# Feasibility exploration of GSH in the treatment of acute hepatic encephalopathy from the aspects of pharmacokinetics, pharmacodynamics, and mechanism

**DOI:** 10.3389/fphar.2024.1387409

**Published:** 2024-06-03

**Authors:** Kangrui Hu, Yexin Xu, Jiye Fan, Huafang Liu, Chanjuan Di, Feng Xu, Linlin Wu, Ke Ding, Tingting Zhang, Leyi Wang, Haoyu Ai, Lin Xie, Guangji Wang, Yan Liang

**Affiliations:** ^1^ Key Lab of Drug Metabolism and Pharmacokinetics, State Key Laboratory of Natural Medicines, China Pharmaceutical University, Nanjing, China; ^2^ Department of Pharmacy, Hebei Chemical and Pharmaceutical College, Shijiazhuang, Hebei Province, China; ^3^ Hebei Zhitong Biopharmaceutical Co., Ltd., Gucheng, Hebei Province, China

**Keywords:** glutathione, acute hepatic encephalopathy, oxidative stress, endoplasmic reticulum stress, iNOS/ATF4/Ddit3

## Abstract

Our previous study highlighted the therapeutic potential of glutathione (GSH), an intracellular thiol tripeptide ubiquitous in mammalian tissues, in mitigating hepatic and cerebral damage. Building on this premise, we posited the hypothesis that GSH could be a promising candidate for treating acute hepatic encephalopathy (AHE). To verify this conjecture, we systematically investigated the feasibility of GSH as a therapeutic agent for AHE through comprehensive pharmacokinetic, pharmacodynamic, and mechanistic studies using a thioacetamide-induced AHE rat model. Our pharmacodynamic data demonstrated that oral GSH could significantly improve behavioral scores and reduce hepatic damage of AHE rats by regulating intrahepatic ALT, AST, inflammatory factors, and homeostasis of amino acids. Additionally, oral GSH demonstrated neuroprotective effects by alleviating the accumulation of intracerebral glutamine, down-regulating glutamine synthetase, and reducing taurine exposure. Pharmacokinetic studies suggested that AHE modeling led to significant decrease in hepatic and cerebral exposure of GSH and cysteine. However, oral GSH greatly enhanced the intrahepatic and intracortical GSH and CYS in AHE rats. Given the pivotal roles of CYS and GSH in maintaining redox homeostasis, we investigated the interplay between oxidative stress and pathogenesis/treatment of AHE. Our data revealed that GSH administration significantly relieved oxidative stress levels caused by AHE modeling via down-regulating the expression of NADPH oxidase 4 (NOX4) and NF-κB P65. Importantly, our findings further suggested that GSH administration significantly regulated the excessive endoplasmic reticulum (ER) stress caused by AHE modeling through the iNOS/ATF4/Ddit3 pathway. In summary, our study uncovered that exogenous GSH could stabilize intracerebral GSH and CYS levels to act on brain oxidative and ER stress, which have great significance for revealing the therapeutic effect of GSH on AHE and promoting its further development and clinical application.

## Graphical Abstract

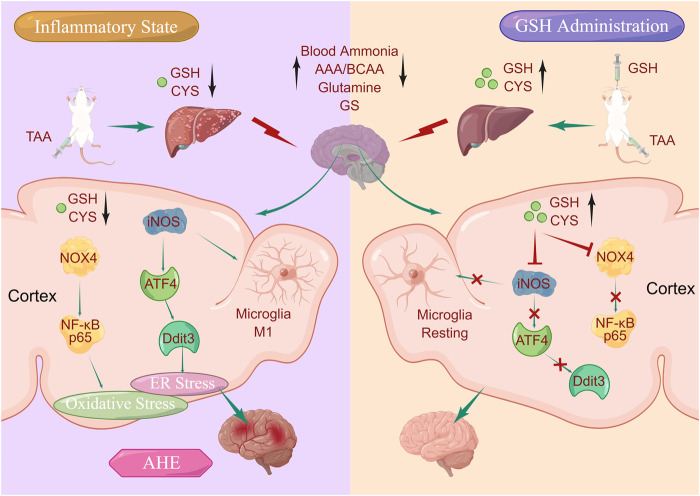

## 1 Introduction

As a subtype of hepatic encephalopathy, acute hepatic encephalopathy (AHE) is characterized by severe neurological disorder caused by acute liver injury, with a mortality rate exceeding 80% (Fu et al., 2019). Clinically, AHE manifests as central nervous system (CNS) dysfunction, including epilepsy, brain edema, brain herniation, and heightened intracranial pressure (Córdoba, 2011; Jayakumar et al., 2011; Wang et al., 2018). Historical studies have shown that arterial and cerebral ammonia levels serve as reliable predictors of the onset and severity of CNS complications in patients with acute liver failure (ALF) (Bosoi et al., 2011; Rajaram and Subramanian, 2018). Ammonia accumulation, a consequence of ALF, breaches the blood-brain barrier and disrupts astrocytic function by altering the expression of key proteins such as glial fibrillary acidic protein, glutamate (GLU), and glycine (GLY) transporters (Butterworth, 2015). Meanwhile, acute ammonia exposure activates N-methyl-D-aspartate (NMDA) receptors and elevates Ca2^+^ level in mitochondria, triggering redox imbalance and enhancing reactive oxygen species production (ROS) (Guazzelli et al., 2020). Moreover, astrocyte-generated excess glutamine under hyperammonemia conditions enters the mitochondrial matrix, where it is metabolized into ammonia through phosphate-activated glutaminase, leading to mitochondrial ROS production and eventually brain edema (Albrecht and Norenberg, 2006). Therefore, oxidative stress and inflammation levels are critical indicators for determining the severity of AHE patients in clinical practice (Butterworth, 2013; Guo et al., 2018). Building upon this framework, the two-hit hypothesis suggests that AHE can be aggravated by inflammation, which enhances the body’s immune response sensitivity and closely correlates with impaired cognitive-motor function (Rodrigo et al., 2010). In recent years, disruptions in amino acid profile have been linked to the onset and progression of AHE, with the branched-chain amino acids/aromatic amino acids (BCAAs/AAAs) ratio serving as a predictive marker for clinical outcomes (Holecek, 2015; Hadjihambi et al., 2018). Therefore, the pathogenesis of AHE is multifaceted, with consensus on a single pathophysiological mechanism responsible for its development.

An increasing number of studies in recent years have consistently identified HE as the most prevalent complication of liver cirrhosis, significantly impacting quality of life, incidence rate, and mortality, thereby exacerbating the medical burden (Rose et al., 2020). However, due to an incomplete understanding of its pathogenesis, clinical treatment of HE is currently restricted to liver transplantation (Prakash and Mullen, 2010; Guo et al., 2018). More treatment options and preventive nursing interventions are needed to decrease the incidence of HE and reduce its socio-economic impact on families and pressure on medical resources. Glutathione (GSH), an endogenous hydrophilic antioxidant, has demonstrated efficacy in shielding from various reactive oxygen species and nitrogen species (Soni et al., 2023). Disruptions in GSH homeostasis have been proven to be closely related to the pathogenesis of various diseases, including obesity, diabetes, ischemic stroke, and other neurodegenerative diseases (Johnson et al., 2012; Nikolaos et al., 2018). Furthermore, increasing evidence suggests that GSH not only detoxifies exogenous substances in the brain but also regulates oxidative defense and intracellular redox homeostasis. Critically, GSH modulates intracerebral cell signaling, protein function, gene expression, and cell differentiation/proliferation in the brain (Aoyama and Nakaki, 2013). Additionally, the amino acid precursors GLU, GLY, and cysteine (CYS), involved in GSH synthesis, play critical roles in the development of CNS diseases. For instance, maintaining strict regulation of cerebral GLU levels is essential, as excessive GLU release (an excitatory neurotransmitter) can induce excitotoxic neuronal damage (Ji et al., 2019). Moreover, GLY, an inhibitory neurotransmitter, has been reported to attenuate brain injury-induced neuronal death by regulating microglia polarization and inhibiting NF-κB p65/Hif-1α signal (Liu et al., 2019). CYS also exhibits antioxidant properties, and its intracerebral concentration is closely associated with neurodegenerative diseases such as Alzheimer’s disease and Parkinson’s disease (Vandiver et al., 2013; Scheltens et al., 2016). In recent years, our research has focused on elucidating the hepatic and cerebral protective effects of GSH. Previous findings indicated that doxorubicin administration decreased GSH levels in mouse liver, and exogenous GSH (5, 50, and 500 mg/kg per day, i.g.) significantly mitigated the cardiotoxicity and hepatotoxicity caused by doxorubicin (Shen et al., 2019). Furthermore, oral GSH (250 mg/kg) exerted direct therapeutic effects in ischemic stroke by stabilizing intracerebral GSH, CYS, and GLU levels, as well as indirectly mitigating intestinal barrier damage (Chen et al., 2020). Additionally, intravenous administration of 10 mg/kg of GSH once a day demonstrated therapeutic efficacy in experimental *Salmonella* meningitis (Guo et al., 2023). Consequently, we investigated the pharmacological activity of GSH on AHE via two administration routes, oral gavage (250 mg/kg) and intravenous injection (10 mg/kg). Notably, the gavage dose of 250 mg/kg in rats aligns with the clinical dosage of GSH (Patel and Hyman, 2020).

Therefore, this study systematically explored the therapeutic potential of exogenous GSH in thioacetamide (TAA)-induced AHE model rats, affirming the feasibility of GSH as a therapeutic agent for AHE through investigations into its pharmacokinetics and molecular mechanisms of AHE treatment. Our results demonstrated that oral GSH administration significantly enhanced the exposure of GSH and CYS in the liver and cortex of AHE rats, as confirmed by targeted Mass Spectrometry Imaging (MSI). Elevated GSH and CYS levels not only improved behavior scores, intrahepatic ALT, AST, inflammatory factors, and amino acid homeostasis but also alleviated brain glutamine accumulation, downregulated glutamine synthetase (GS) and reduced taurine exposure. Moreover, GSH administration effectively mitigated oxidative stress induced by AHE modeling by suppressing the expression of NADPH oxidase 4 (NOX4) and NF-κB P65. Furthermore, GSH administration regulated excessive endoplasmic reticulum (ER) stress caused by AHE modeling through the inducible nitric oxide synthase (iNOS)/activating transcription factor 4 (ATF4)/DNA damage-induced transcript 3 (Ddit3) pathway.

## 2 Material and methods

### 2.1 Chemicals and reagents

GSH standard was purchased from Shandong Jincheng Biopharmaceutical Co., Ltd. (Zibo, Shangdong, China). The standards of CYS, CYS-GLY, GLU, GLY, captopril (CAP), N-ethylmaleimide (NEM), and ^13^C-glutamine were purchased from Sigma-Aldrich Co. (St. Louis, MO, United States). Test kits for blood ammonia, glutathione disulfide (GSSG), GSH, Malondialdehyde (MDA), Superoxide dismutase (SOD), ROS, and BCA protein were purchased from Shanghai Beyotime Biotechnology Research Institute, China (Shanghai, China). ALT and aspartate AST assay kits were purchased from Nanjing Jiancheng Bioengineering Institute (Nanjing, China). Interleukin-6 (IL-6), interleukin-10 (IL-10), tumor necrosis factor-alpha (TNF-α), interleukin-1β (IL-1β) assay kits were purchased from Shanghai Excell Biological Technology Co., Ltd. (Shanghai, China). Primers of NOX4, 3-phosphate Glyceraldehyde dehydrogenase (GADPH), Ddit3, and ATF4 were purchased from Abcam Co., Ltd. (Cambridge, UK). NF-κB p65 antibodies were purchased from Cell Signaling Technology (Danvers, MA, United States). RNAiso Plus, Prime Script RT Master Mix, and SYBR Premix Ex Taq (Perfect Real Time) were purchased from Takara Biotechnology Co., Ltd. (Shiga, Japan). NOX4, Ddit3, and ATF4 antibody were purchased from Abcam Ltd. (Cambridge, UK). NF-κB antibody was purchased from Cell Signaling Technology (Danvers, MA, United States).

### 2.2 Animals and treatments

Male Sprague-Dawley (SD) rats (aged 8–9 weeks, 200–220 g) were purchased from the Sipper-BK laboratory animal CO., Ltd. (Shanghai, China) (approval number: SCXK (HU) 2013–0016), by guidelines of National Institutes of Health Guide for the Care and Use of Laboratory Animals. The rats were housed under controlled conditions (25°C, 55%–60% humidity, and 12 h light/dark cycle) with free access to laboratory food and water. All studies were conducted strictly with animal care regulations and guidelines and were approved by the China Pharmaceutical University Animal Care and Use Committee. The rats were habituated to the facilities for a week before the experiment.

To construct an AHE rat model, we injected TAA intraperitoneally into the rats at a dose of 250 mg/kg for three consecutive days. After AHE modeling, rats in the GSH-dosed group were intragastrically administrated with GSH at a dose of 250 mg/kg for two consecutive days. Besides, an individual experiment also investigated the therapeutic effect of intravenous administration of GSH. After AHE modeling, rats in the GSH-dosed group were intravenously administered with GSH at 10 mg/kg for two consecutive days. All rats were euthanized after 2 days of GSH treatment, then blood and tissues were collected.

### 2.3 Neurological assessments

The behavioral changes observed in rats were categorized into different levels to assess their neurological status. Here are the descriptions of each level:

Level 0: Rats exhibit normal or regular physical activity, display a startled response to sound stimuli, and demonstrate the presence of the righting reflex, indicating normal neurological function.

Level 1: Rats display mild drowsiness characterized by reduced spontaneous motor activity. However, they still exhibit a normal regular response to stimuli and retain the righting reflex.

Level 2: Rats show a lack of spontaneous motor activity but may exhibit some movements, reactions, and righting reflexes when aroused. Despite reduced activity, they maintain some level of responsiveness.

Level 3: Rats remain stationary and show reluctance to move. Their reactions to stimuli are significantly reduced, and they may demonstrate flipping reflexes when manipulated.

Level 4: Rats remain stationary with no observable reaction to stimuli. They do not display the righting reflex, indicating severe neurological impairment.

### 2.4 Histological analysis

The same part of liver tissue slices from different groups were fixed in 4% Paraformaldehyde and then stained with Picro-Sirius red solution according to standard technique. The tissue sections were washed with acidified water and counterstained with Carazzi’s hematoxylin.

### 2.5 Biochemical analysis of plasma and tissues

The levels of blood ammonia, ALT, AST in plasma, the levels of intrahepatic IL-6, IL-10, and the levels of intracerebral SOD, ROS, IL-6, IL-10, TNF-α were determined using the corresponding commercial biochemical kits following the manufacturer’s instructions. The results were calibrated using protein concentration.

### 2.6 Quantitative analysis of GSH, CYS, CYS-GLY, GLU, GLY, glutamine, and taurine in tissues

Our previous study has established an LC-MS/MS method for the quantitative analysis of GSH, CYS, and CYS-GLY in the biological matrix (Chen et al., 2020). It used NEM derivatization according to established protocol (Giustarini et al., 2013). Briefly, 10 mg fresh tissues were homogenized with 100 μL buffer solution (6 mg/mL Tris, 0.2 mg/mL serine, 1.24 mg/mL boric acid, 4 μg/mL acivicin, and 7.76 mg/mL NEM). After adding 10 µL of internal standard solution (CAP, 5 μg/mL) into 50 µL of tissue homogenate, derivation by NEM was lasted under the light proof environment for 1 h, followed by adding 200 μL of methanol to precipitate proteins. After vortex-mixing for 5 min and centrifuging at 30,000 g for 10 min at 4°C, 50 μL supernatant was collected and analyzed using the LC-MS/MS system (SCIEX 6500, MA, United States) in positive ionization mode. The injection value was 5 μL and chromatographic separation was performed on a Sepax Bio-ODS SP column (5 μm, 4.6 mm × 150 mm). Mobile phase A was H_2_O with 2 mM ammonium formate and 0.1% formic acid. Mobile phase B was methanol. The gradient elution was set as follows: 10% B at 0 min and held for 0.2 min, increased to 50% B at 0.4 min and held for 3.8 min, then decreased to 10% B at 4.5 min followed by 5 min for equilibration. The source conditions were set as follows: ion source gas 1, 60 psi; ion source gas 2, 50 psi; collision gas, 6 psi; curtain gas, 10 psi; ion-spray voltage, 4,500 V; source temperature, 550°C. The details of the optimized multiple reaction monitoring (MRM) parameters were summarized in Supplementary Table S1.

Quantitative analysis of GLU, GLY, glutamine, and taurine in tissues was performed according to the following procedure. Fresh tissue (10 mg) was homogenized with 100 μL of H_2_O, followed by adding 400 μL of methanol solution containing internal standard (^13^C-glutamine, 15 μg/mL). After centrifugation at 40,000 g for 10 min, 300 μL supernatant was evaporated and reconstituted with 150 μL H_2_O. Then the GLU, GLY, glutamine and taurine were analyzed using an LC-Q-TOF-MS system (SCIEX 5600, MA, United States) in negative ionization mode, and chromatographic separation was performed on a Waters Bridge Amide column (3.5 μm, 4.6 × 100 mm). Mobile phase A was H_2_O with 5 mM ammonium acetate and 5% acetonitrile, and pH was adjusted to 9 with aqueous ammonia. Mobile phase B was acetonitrile. The gradient elution was as follows: 85% B at 0 min and held for 3 min, decreased to 30% B at 6 min, decreased to 2% B at 15 min and maintain for 3 min, then increased to 85% B at 19 min followed by balancing for 5 min. The MS parameters were set as follows: nebulizer gas, 50 psi; heater gas, 60 psi; curtain gas, 30 psi; declustering potential, 70 V; ion-spray voltage, −4.5 kV; source temperature 500°C. The mass scanning ranges of MS^1^ and MS^2^ were set at 100–1,000 and 50–1,000, respectively. CE value was set at 30 V with a spread of 20 V. The data were processed by MultiQuant 2.0 Software (Sciex, Concord, Ontario, Canada).

### 2.7 Determination of MDA and GSSG

As to MDA, thiobarbitoric acid assay is the most commonly used method for MDA determination (Janero and Burghardt, 1988), and we used the MDA Assay Kit purchased from Shanghai Beyotime Biotechnology Research Institute (Cat. No. S0131S). Based on the kit instruction, firstly, tissues of rat brain cortex were homogenized with PBS (the proportion of tissue weight to PBS is 10%). After centrifugation at 12,000 g for 10 min, 100uL of the supernatant were collected as test samples. Next, added 100 µL of MDA standards (1, 2, 5, 10, 20, 50 µM) or test samples to 1.5 mL tubes, then added 200 µL of MDA working solution (containing 0.0925% thiobarbitoric acid), heating in a boiling water bath for 15 min. The water bath was cooled to room temperature, followed by centrifugation at 1,000 g for 10 min 200 μL of the supernatant were transferred to a 96-well plate, and absorbance was measured at 532 nm using a spectrophotometer. Finally, calculated the concentration of MDA according to the standard curve.

As to GSSG, we used the GSSG Assay Kit purchased from Shanghai Beyotime Biotechnology Research Institute (Cat. No. S0053), the theory of which was based on the established enzymatic recycling method (Rahman et al., 2007). According to the kit instruction, specifically, tissues of rat cortex were frozen with liquid nitrogen and then ground into powder. For every 10 mg of ground tissue powder, 100 uL of protein removal reagent M solution was added and homogenized. After being placed at 4°C for 10 min, homogenate was centrifuged at 12,000 g for 10 min, and 100 uL of supernatant were collected as test samples. Next, added 100 µL of GSSG standards (0.5, 1, 2, 5, 10, 15 μM) or test samples to 1.5 mL tubes, then added 20 μL of GSH Removal Buffer, and vortex-mixed immediately. Next, added 4 μL of GSH Removal Reagent to every 100 μL of sample, and vortex-mixed immediately. After incubating at 25°C for 60 min, measured absorbance at 412 nm and calculated the concentration of GSSG according to the standard curve.

### 2.8 DESI-MSI analysis of GSH in liver and brain

Tissues of liver and brain were sectioned at a 10 μm thickness by using a Leica CM1950 cryostat (Leica Microsystem Ltd.) and mounted onto glass slides. Targeted MSI experiments were carried out on a DESI-MSI platform equipped with a DESI XS source and a tandem quadrupole mass spectrometers (Waters, Milford, MA, United States). The spray solvent was acetonitrile/water/formic acid (98:2:0.01, v/v/v, 2 μL/min). The MRM parameters for each analyte were summarized in Supplementary Table S2.

### 2.9 Quantitative analysis of amino acids in plasma

Our previous study has established a LC-MS/MS method for the quantitative analysis of amino acids in biological matrix (Shen et al., 2021). Ascorbic acid (20 mM) was added to 30 mg of the rat striatum. The mixtures were homogenized and sonicated in an ice bath. Then, 500 μL of ice-cold acetonitrile containing 200 ng of 2,5-dihydroxy benzoic acid was added to the homogenate. After drying under vacuum, 50 μL of borate buffer and 50 μL of benzoyl chloride were added for derivatization. The chromatographic separation was performed on an XBridge^®^ Amide column (3.5 µm × 4.6 mm × 100 mm, Waters). The mobile phase consisted of solvent A (0.2% formic acid and 5.0 mM ammonium formate in water) and solvent B (acetonitrile). The MS was operated using the LC-MS/MS system (SCIEX 6500, MA, United States) in positive mode. The MS parameters were set as follows: ion source gas1, 55; ion source gas 2, 50; curtain gas, 20; CAD gas, medium; ion spray voltage, 5.0 kV; source temperature, 550 °C. Supplementary Table S3 summarized the MRM monitoring conditions for each analyte.

### 2.10 Real-time quantitative reverse transcription polymerase chain (Q-PCR) analysis of inflammatory factors, NOX4, iNOS, Ddit3, and ATF4

Total RNA was isolated from rat cortex with RNAiso plus and Q-PCR analyses were performed using SYBR Premix Ex Taq following the manufacturer’s protocols. The primers used were summarized in Supplementary Table S4. The Q-PCR cycle conditions were at 95°C for 30 s, followed by 40 cycles at 95°C for 5 s and at 60°C for 30 s. Then the amplification specificity was evaluated with melting curve analysis.

### 2.11 Western blot

Protein levels of NADPH Oxidase 4, NF-κB p65, Ddit3, and ATF4 in rat cortex were measured by Western blot analysis. Equal loading of proteins was verified by immunoblotting of GAPDH. Gel-Pro analyzer was used for the semi-quantitative analysis of the proteins obtained from gel imaging system.

### 2.12 Statistical analysis

All the results were shown with mean ± SD of 6–8 mice per group. Data were analyzed by using two-tailed t-test, or one-way ANOVA test with Student-Newman-Keuls test for comparison of two groups. Values of *p* < 0.05 were considered statistically significant.

## 3 Results

### 3.1 Hepatoprotective effect of exogenous GSH on AHE rats

**FIGURE 1:**
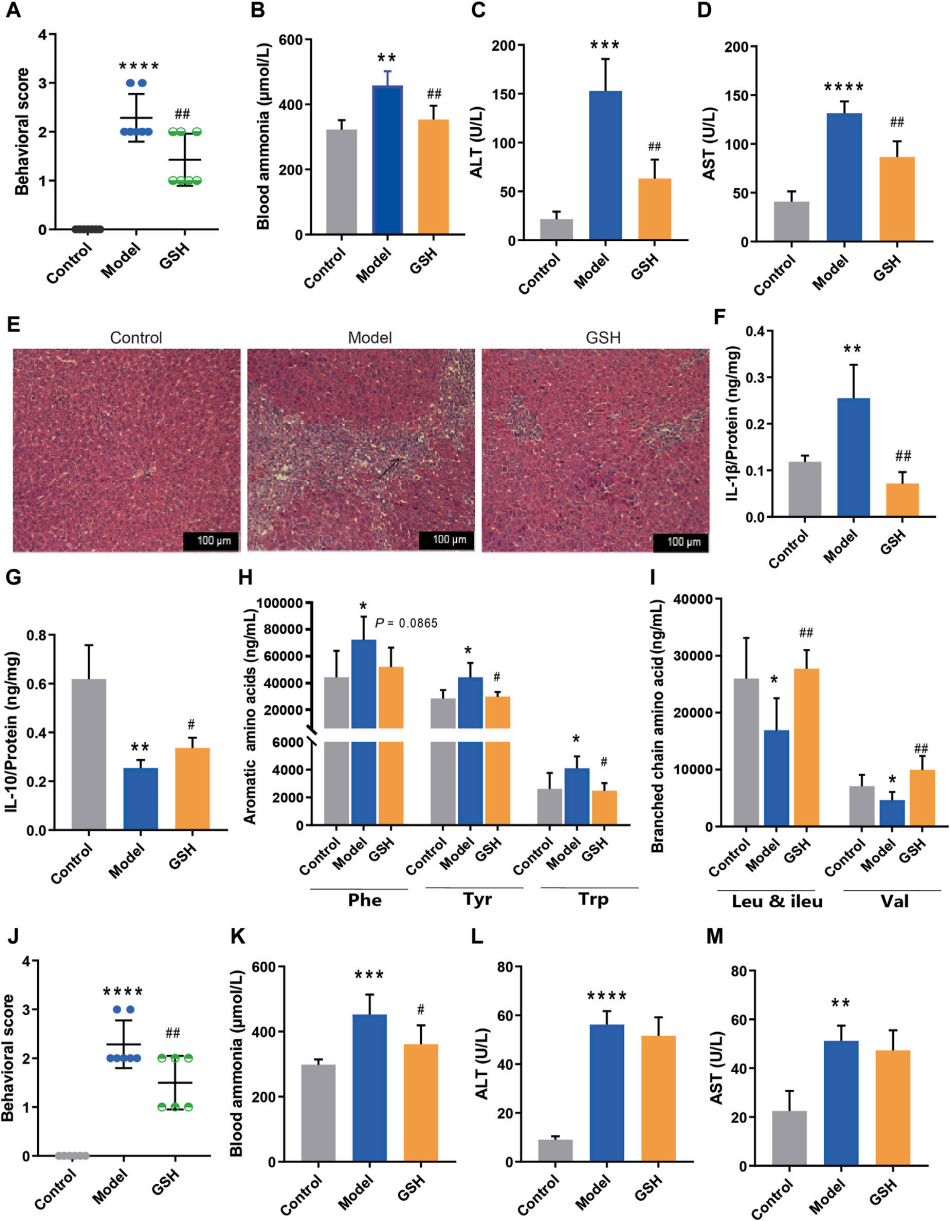
Hepatoprotective effect of exogenous GSH on AHE rats. Effect of oral administration of GSH on **(A)** behavioral scores, **(B)** blood ammonia, **(C)** serum ALT, **(D)** serum AST, **(E)** pathological sections of liver, **(F)** hepatic IL-1β, **(G)** hepatic IL-10, **(H)** aromatic amino acids in serum, **(I)** branched-chain amino acids in serum; Effect of intravenous administration of GSH on **(J)** behavioral scores, **(K)** blood ammonia, **(L)** serum ALT, **(M)** serum AST.

Previous research has unequivocally validated the pivotal role of GSH in conferring significant protection against liver and brain injuries. To further enhance the clinical utility of GSH, we investigated the therapeutic effect of GSH on AHE in rats via both oral and intravenous administration modes. Firstly, we investigated the hepatoprotective effect of exogenous GSH on AHE rats. In this process, TAA-induced AHE rats were orally administered GSH or physiological saline for two consecutive days, followed by euthanasia under anesthesia 24 h after the final administration. Behavioral assessments revealed significantly elevated scores in AHE rats compared to the control group, with oral GSH administration markedly ameliorating the scores (Figure 1A). Furthermore, oral GSH administration effectively mitigated the substantial rise in blood ammonia concentration caused by TAA (Figure 1B). AHE modeling led to notable hepatic injury, characterized primarily by a significant elevation in serum ALT and AST levels, which were dramatically reduced by oral GSH administration (Figures 1C,D). Subsequently, Hematoxylin and eosin (H&E) staining was used to estimate the liver histopathological changes in different groups. As shown in Figure 1E, AHE rats exhibited severe inflammatory cell infiltration in the liver accompanied by tissue hemorrhage, which was effectively alleviated by oral GSH administration. AHE modeling significantly increased pro-inflammatory factor IL-1β protein levels and downregulated anti-inflammatory factor IL-10 in rat liver (Figure 1F, G). Oral GSH administration extensively modulated intrahepatic inflammatory factors in AHE rats to the normal controls. Therefore, exogenous GSH could exert significant hepatoprotective effects in AHE rats.

According to prior reports, amino acid profiles dysregulation is impilicated in the onset and progression of AHE, with the BCAAs/AAAs ratio serving as a clinical prognostic indicator (Holecek, 2015; Hadjihambi et al., 2018). To investigate the influence of oral GSH on AHE, concentrations of AAAs (phenylalanine (Phe), tyrosine (Tyr), and tryptophan (Trp)) and BCAAs (leucine (Leu) and valine (Val)) in rat plasma were quantitatively analyzed using the LC-MS/MS assay previously established (Shen et al., 2021). As shown in Figure 1H, I, AHE modeling led to a noticeable elevation in AAAs and a significant reduction in BCAAs. Oral GSH administration restored AAAs and BCAAs in AHE rat plasma to conventional levels, thereby maintaining amino acid homeostasis.

The therapeutic effect of intravenous GSH injection in AHE was also investigated in rats. Notably, intravenous GSH administration at a dose of 10 mg/kg significantly improved the behavioral scores of AHE rats (Figure 1J). Additionally, intravenous GSH administration effectively mitigated the notable increase in plasma ammonia concentration caused by TAA (Figure 1K). AHE modeling resulted in a significant elevation in serum ALT and AST levels. However, intravenous GSH administration of GSH did not significantly reduce the levels of ALT and AST in the serum of AHE rats (Figure 1L, M). Thus, the hepatoprotective effect of intravenous administration of 10 mg/kg of GSH on AHE rats was considerably lower than that of oral administration of 250 mg/kg of GSH.

### 3.2 Cerebral protection of exogenous GSH on AHE rats.

**FIGURE 2:**
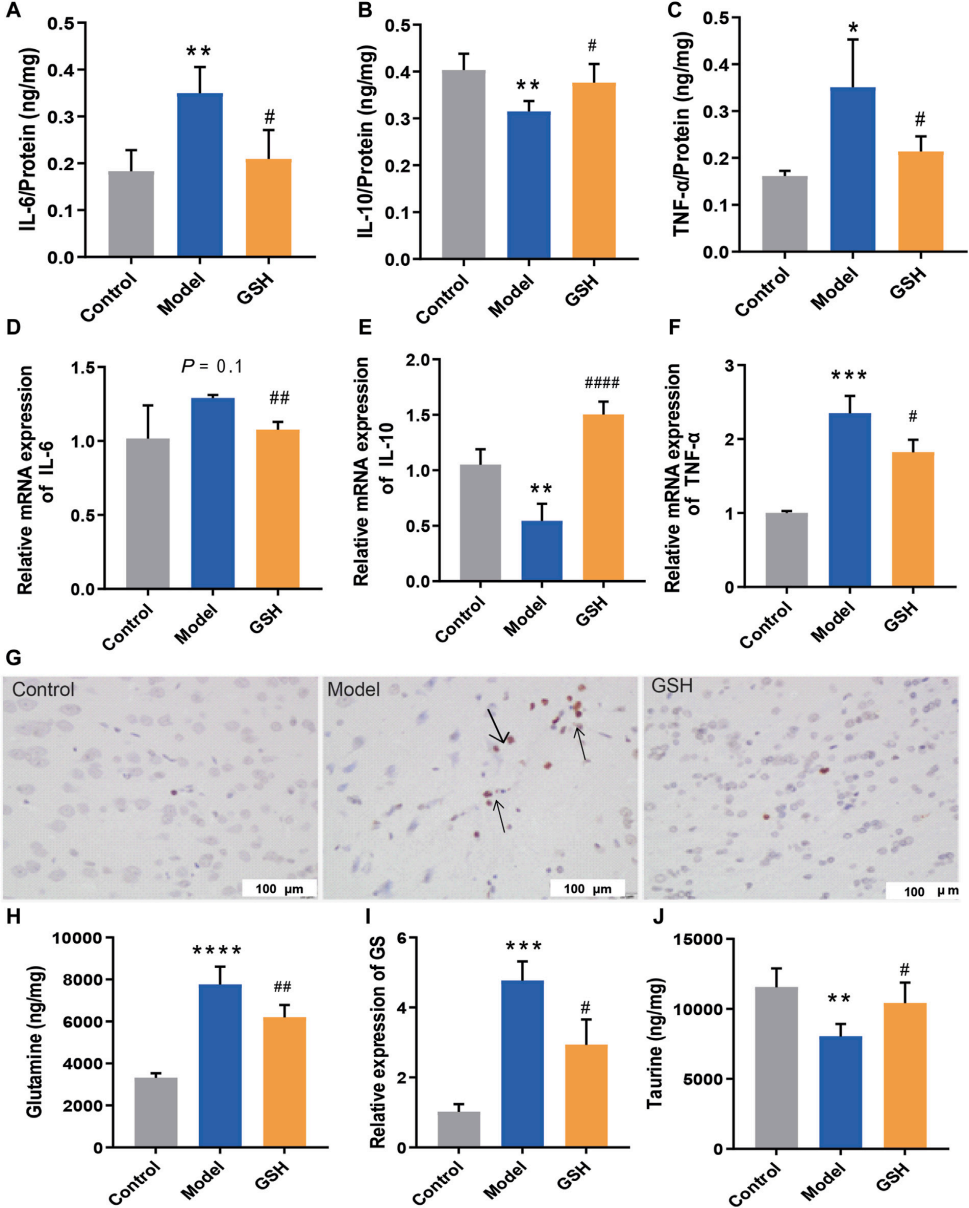
Cerebral protection of oral administration of GSH on AHE rats. Protein expression of **(A)** IL-6, **(B)** IL-10, **(C)** TNF-α; mRNA expression of **(D)** IL-6, **(E)** IL-10, **(F)** TNF-α; **(G)** TUNEL staining of rat cortex; **(H)** Glutamine concentrations, **(I)** relative expression of GS, **(J)** taurine exposure in rat cortex.

After determining the hepatoprotective effect of GSH, we further investigated its cerebral protective effects on AHE rats. The protein and mRNA levels of intracerebral inflammatory factors (IL-6, TNF-α, and IL-10) were determined using Elisa and Q-PCR assays. In this process, we compared the alterations of inflammatory factors across different brain regions (hippocampus, striatum, and cortex) and identified a strong correlation between inflammatory factors in the cortex and the onset/treatment of AHE. Notably, AHE modeling significantly upregulated the pro-inflammatory factors IL-6 and TNF-α in the rat cortex, while down-regulating the anti-inflammatory factor IL-10. Oral GSH administration effectively modulated intracerebral inflammatory factors in AHE rats towards the conventional levels (Figure 2A–F). Additionally, cortical tissues from different groups were subjected to TUNEL staining. AHE model rats showed a substantial number of stained condensed nuclei, indicating significant cell apoptosis. GSH administration notably reduced the number of stained condensed nuclei and attenuated cell apoptosis (Figure 2G). Intracerebral glutamine, a toxic substance, was quantified using LC-MS, revealing a marked accumulation in AHE-induced rats. Conversely, oral GSH administration significantly alleviated glutamine accumulation (Figure 2H). Given that GS catalyzes the synthesis of glutamine from ammonia, we further investigated the effect of GSH on GS enzyme activity. Evidently, AHE modeling markedly upregulated GS expression, while GSH administration significantly reversed the upregulation (Figure 2I). Moreover, we assessed the concentration of taurine, which maintains sodium ion balance in the cortex of conventional AHE model and GSH-treated rats. The results demonstrated that GSH administration significantly reversed the reduction in intracerebral taurine exposure caused by AHE modeling (Figure 2J). Therefore, exogenous GSH exerted significant cerebral protective effects in AHE rats.

### 3.3 Regulation of GSH on the exposure of GSH-derived ingredients

**FIGURE 3:**
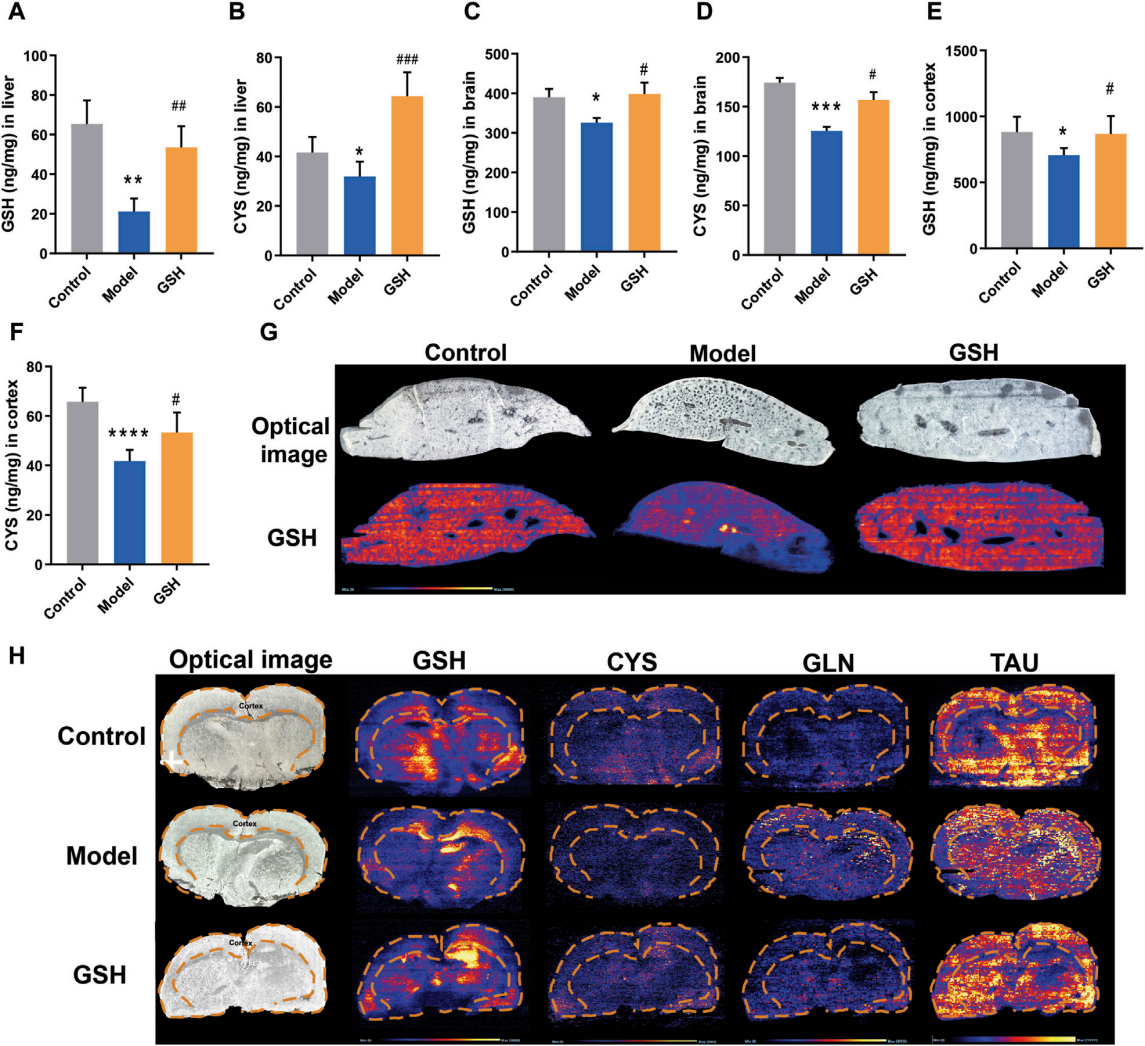
Influence of oral administration of GSH on the distribution of GSH and CYS in the brain and liver of AHE rats. Exposure of **(A)** GSH and **(B)** CYS in rat liver; Exposure of **(C)** GSH and **(D)** CYS in rat brain; Exposure of **(E)** GSH and **(F)** CYS in rat cortex. **(G)** MSI for GSH, CYS, glutamine (GLN), and taurine (TAU) in mouse brain using DESI XS. **(H)** MSI for GSH in mouse liver.

In general, GSH could be hydrolyzed to cysteine-glutathione (CYS-GLY), glutamate (GLU), CYS, and GLY *in vivo* (Chen et al., 2020). To further elucidate the pharmacological substances contributing to the therapeutic effects on AHE, the exposure of GSH-derived ingredients (GSH, CYS, GLU, GLY, and CYS-GLY) in target tissues was determined using LC-MS/MS and LC-Q-TOF/MS systems. As shown in Figure 3A–D, AHE modeling led to a considerable reduction in GSH and CYS exposure in the rat liver and brain, whereas oral GSH administration significantly increased intracellular GSH and CYS levels. Furthermore, the concentrations of GSH and CYS across different brain regions, including cortex, hippocampus, and striatum, were quantitatively analyzed to further determine the specific action sites of GSH. As shown in Figure 3E, F, we observed significantly lower exposure of GSH and CYS in the cortex of AHE rats compared to controls, with oral GSH administration of GSH effectively elevating their levels in this region. However, neither AHE induction nor GSH administration had a discernible effect on GSH and CYS exposure in the hippocampus and striatum of rats (Supplementary Figure S1G–J). DESI-MSI analysis further supported our findings, demonstrating that GSH levels decreased in the liver and cerebral cortex due to AHE modeling but were restored by oral GSH administration. Additionally, the MSI results of CYS, glutamine, and taurine were consistent with previous data (Figure 3G, H). We also investigated the influence of AHE modeling and GSH administration on the targeted exposure of CYS-GLY, GLY, and GLU. Our results indicated no significant differences in intrahepatic CYS-GLY, GLY, and GLU levels among control, AHE, and GSH-treated rats (Supplementary Figure S1A–C). Similarly, neither AHE induction nor GSH administration had a pronounced effect on the intracerebral exposure of CYS-GLY and GLY, although there was some regulatory effect on GLU (Supplementary Figure S1D–F). Following 2 days of intravenous GSH administration, intrahepatic GSH and CYS levels increased to a certain extent, although there was no significant difference between the GSH-treated and AHE rats (Supplementary Figure S2A, B). Similarly, intracortical GSH and CYS levels were significantly lower in AHE rats than in controls, with intravenous GSH administration effectively increasing their content in the cortex (Supplementary Figure S2C, D). However, intravenous GSH administration of GSH had no obvious effect on GSH and CYS exposure in the hippocampus and striatum of rats (Supplementary Figure S2E–H).

**FIGURE 4:**
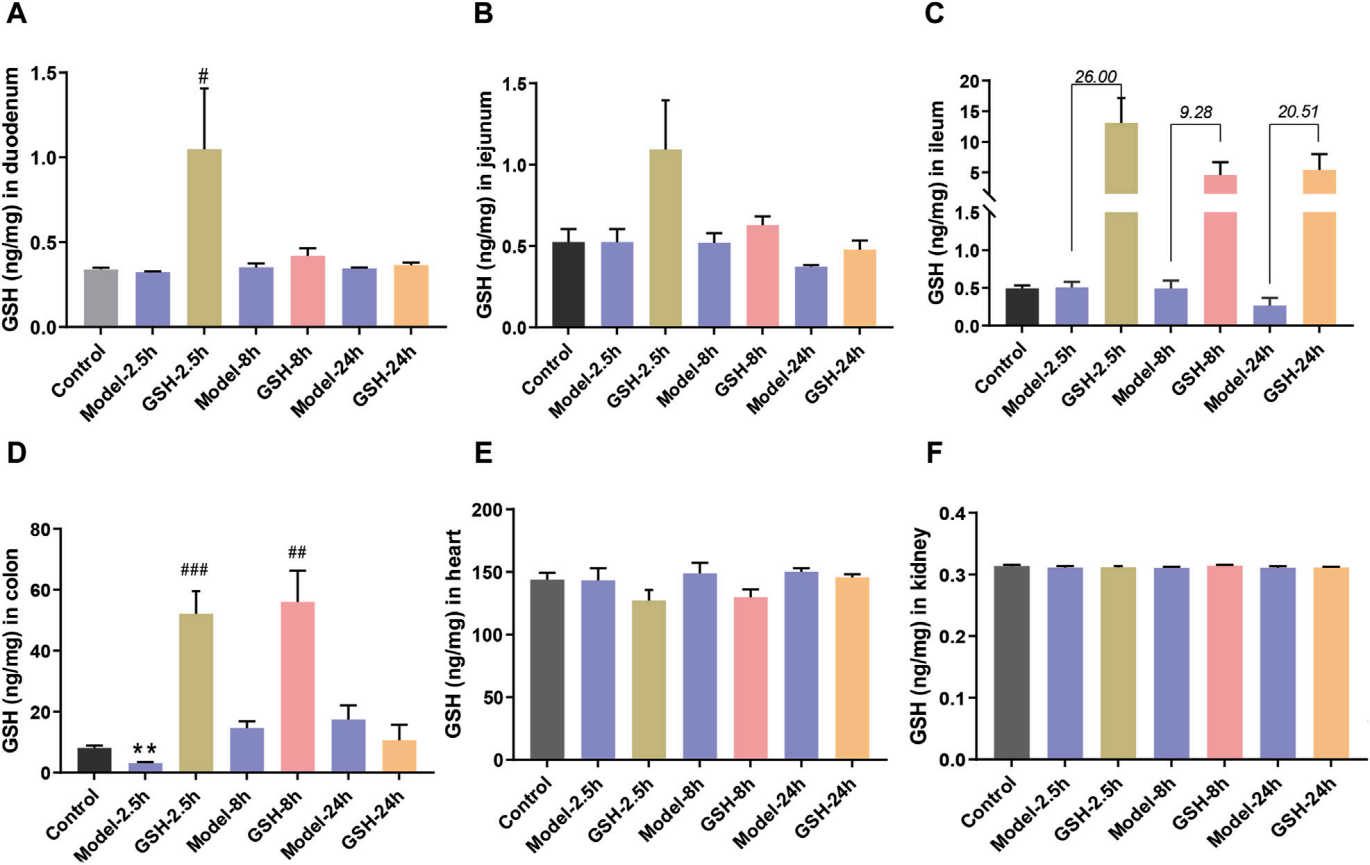
Influence of oral administration of GSH on the distribution of GSH in the tissues of AHE rats. Exposure of GSH in **(A)** duodenum, **(B)** jejunum, **(C)** ileum, **(D)** colon, **(E)** heart, **(F)** kidney.

To further delineate the action sites of GSH, we studied its distribution in other tissues of AHE rats. Concentrations of GSH in various rat tissues (heart, kidneys, duodenum, jejunum, ileum, and colon) were quantitatively analyzed at 2.5, 8, and 24 h after administration. As shown in Figure 4, AHE modeling had no obvious effect on GSH concentrations in the heart, kidney, and intestines, while oral GSH administration significantly enhanced the exposure of GSH in the intestine of AHE rats. Particularly, GSH exposure in the ileum increased substantially after oral GSH administration. Similarly, oral GSH administration also greatly elevated GSH exposure in rat colon. Conversely, oral GSH administration had no significant influence on GSH levels in the heart and kidney of AHE rats. Intratissue concentrations of GSH in AHE rats after intravenous GSH administration were also measured, revealing no significant effect on GSH exposure in heart, kidney, and intestinal segments (Supplementary Figure S3).

### 3.4 Regulation of GSH on oxidative stress

**FIGURE 5:**
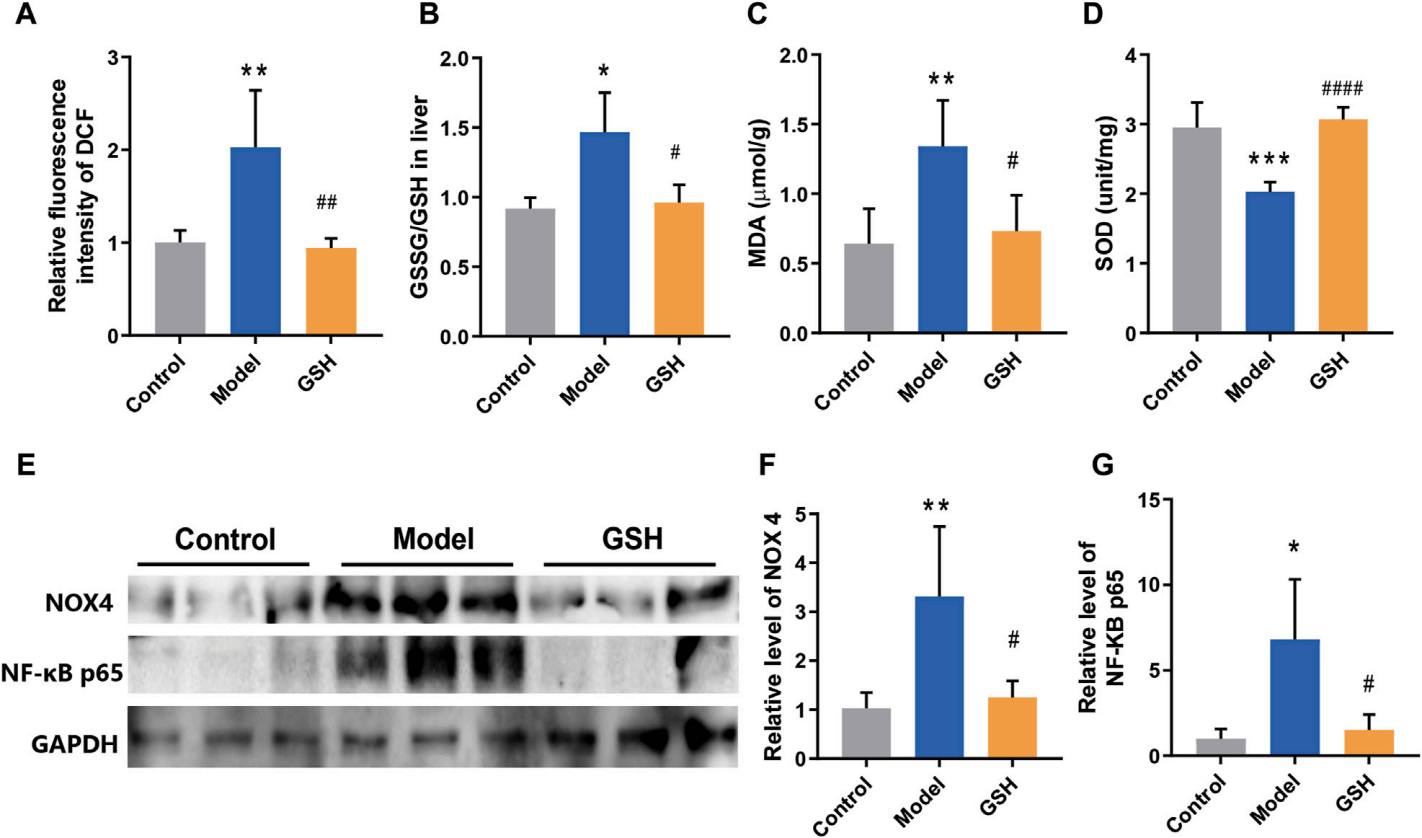
Regulation of GSH on oxidative stress. **(A)** ROS levels, **(B)** GSSG/GSH ratio, **(C)** MDA levels, **(D)** SOD levels, **(E–G)** protein expression of NOX4 and NF-κB P65 in rat cortex.

Oxidative stress is a ubiquitous phenomenon in the body and its exacerbation is associated with several diseases (Simicic et al., 2022). However, the intricate interplay between oxidative stress regulation and the pathophysiology/treatment of AHE remains largely unexplored. Therefore, we endeavored to elucidate the molecular mechanism underlying GHS treatment for AHE by examining its effects on cortical oxidative stress in rats. Firstly, the ROS level in rat cortex was detected by DCFH-DA fluorescent probes. The results revealed a marked increase in ROS following AHE modeling, which were effectively restored to baseline levels upon GSH administration (Figure 5A). Moreover, the GSSG/GSH ratio has consistently been used to indicate oxidative stress in cells and tissues (Skowrońska and Albrecht, 2013). Herein, the concentrations of GSSG and GSH in rat cortex were measured, and the results demonstrated that AHE modeling led to a noticeable increase of GSSG/GSH ratio, which was substantially reduced following GSH administration (Figure 5B). Similarly, GSH administration mitigated the increase in MDA levels caused by AHE modeling (Figure 5C). Additionally, SOD, a crucial antioxidant enzyme responsible for scavenging superoxide anion free radicals, was found to be downregulated in the AHE rat cortex. GSH administration effectively reversed this downregulation of SOD (Figure 5D).

NOX4, a major source of ROS, has been identified in brain damage. However, its role in regulating the development and treatment of AHE remains elusive. Thus, the protein expression of NOX4 in control, AHE, and GSH-treated groups were measured. The results demonstrated a significant upregulation of NOX4 expression following AHE induction, which was reversed upon GSH administration (Figure 5E, F). Furthermore, NF-κB, which modulates gene expression in various cellular processes including immune response, cell proliferation, and stress response, has been implicated in inflammation and oxidative stress modulation (Skowrońska and Albrecht, 2013). Notably, the protein expression of NF-κB P65 in the cortex of rats showed a significant upregulation post-AHE modeling, which was effectively reversed by GSH administration (Figures 5E, G).

### 3.5 Regulation of GSH on ER stress

**FIGURE 6:**
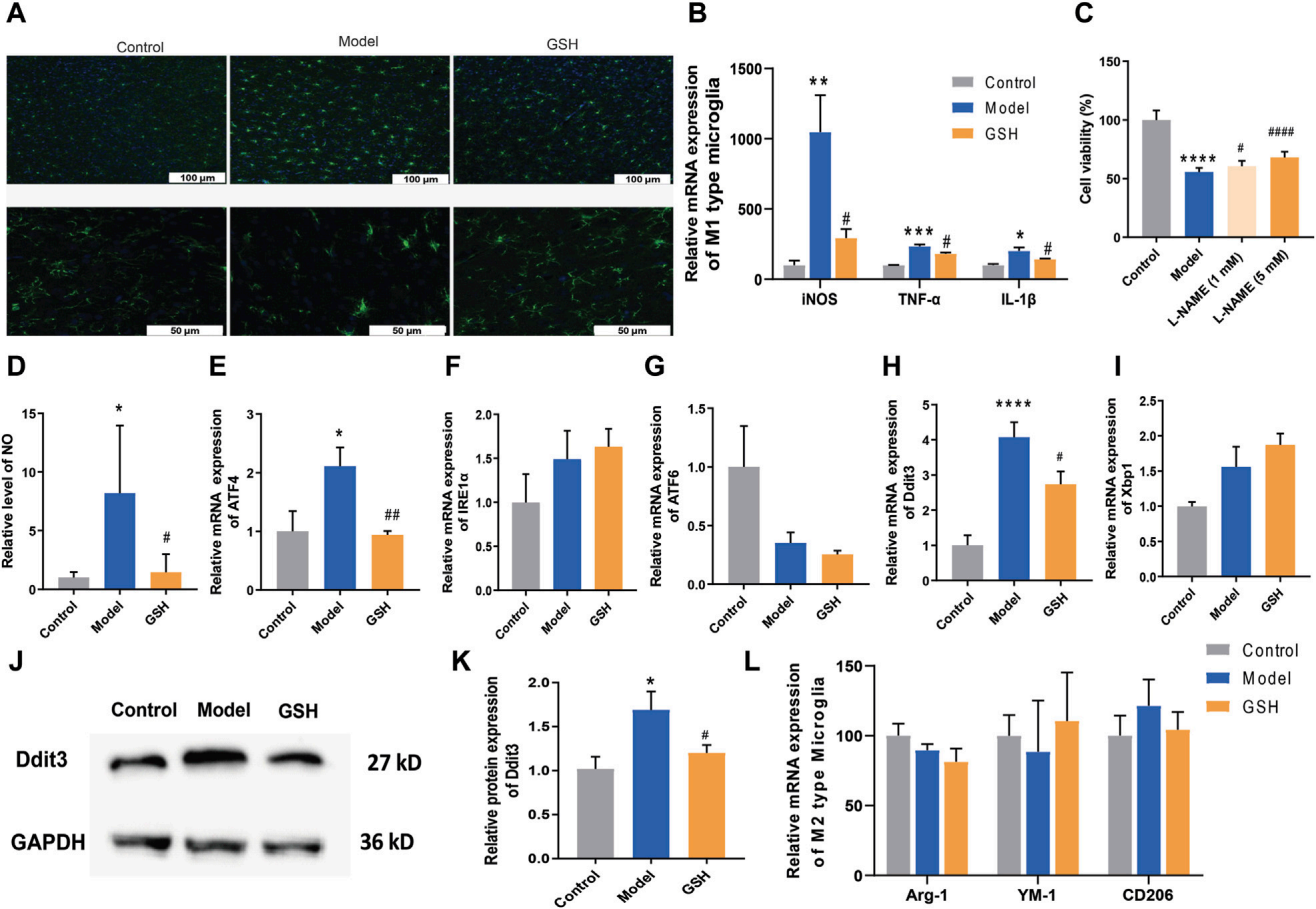
Regulation of GSH on endoplasmic reticulum stress. **(A)** Immunofluorescence staining on microglia; **(B)** Relative expression of M1 type of microglia; **(C)** Cell viability; **(D)** Content of NO in rat cortex; Relative mRNA expression of **(E)** ATF4, **(F)** IRE1α, **(G)** ATF6; Relative mRNA expression of **(H)** Ddit3 and **(I)** Xbp1; **(J and K)** Relative protein expression of Ddit3; **(L)** Relative expression of M2 type of microglia.

Microglia, the foremost active defense cells in the CNS, usually maintain a quiescent state. Herein, we performed immunofluorescence staining on microglia in rat cortex to observe their morphological changes. As shown in Figure 6A, the microglia in the rat cortex became active after AHE modeling, characterized by increased density, rounded shape, and diminished branching. Conversely, in the cortex of rats administered with GSH, microglial density decreased, and the number of branches increased, indicating a significant inhibition of microglial activation by GSH. Microglial activation can be broadly categorized into classical (M1: pro-inflammatory) or alternative (M2: anti-inflammatory) phenotypes. Generally, M1 activation state is associated with the release of pro-inflammatory cytokines, with genes such as iNOS, TNF-α, and IL-1β being key markers. Subsequently, we measured the expression of M1 microglia-related genes in the rat cortex of the control, AHE, and GSH-treated groups. The results demonstrated that iNOS, TNF-α, and IL-1β levels were significantly elevated after AHE modeling and greatly reduced by GSH administration (Figure 6B). Notably, iNOS exhibited the highest degree of regulation following both AHE modeling and GSH administration. To further elucidate the role of iNOS in AHE treatment, we employed the iNOS inhibitor N(ω)-nitro-L-arginine methyl ester (L-NAME) and observed its impact on cell viability in a NH_4_Cl-simulated human malignant cell line U251. L-NAME could significantly enhance the cell viability in a dose-dependent manner (Figure 6C). Additionally, we measured the NO conctent in the rat cortex of different groups. It was found that GSH administration significantly reduced the abnormal elevation of NO induced by AHE modeling (Figure 6D).

Since apoptosis by the ER stress pathway could be enhanced by the iNOS, we measured ER stress-related genes (ATF4, IRE1α and ATF6) to further verify the crucial role of iNOS in AHE occurrence and treatment. As shown in Figures 6E–G, GSH treatment significantly reversed the upregulation of ATF4 caused by AHE modeling, while there was no obvious effect on the expression of IRE1α and ATF6. We then investigated the effects of AHE modeling and GSH administration on the target proteins (Ddit3 and Xbp1) of ATF4. Clearly, GSH treatment significantly reversed the upregulation of Ddit3 caused by AHE modeling, with no notable effect on Xbp1 mRNA expression (Figures 6H–K). Therefore, GSH administration might mitigate AHE by regulating ER stress through the iNOS/ATF4/Ddit3 pathway.

M2 activation state expresses anti-inflammatory cytokines, arginase-1 (Arg1), transforming growth factor β1 (TGFβ1), CD206 and Chitinase-3-like-3 (YM-1 in rodents) (Skowrońska and Albrecht, 2013). We investigated the influence of AHE modeling and GSH administration on the expression of Arg1, CD206 and YM-1 in the rat cortex. However, our results indicated no significant difference in the levels of M2 microglia-related genes among the groups, suggesting that neither AHE modeling nor GSH administration induced alternative activation of microglia (Figure 6L).

## 4 Discussion

GSH, an essential intracellular thiol tripeptide present in mammalian tissues, serves as a critical nucleophilic scavenger and enzyme-catalyzed antioxidant, particularly in response to electrophilic/oxidative tissue damage (Soni et al., 2023). Its deficiency has been implicated in various diseases, including cancer, neurodegenerative diseases, cystic fibrosis, viral infections including HIV-1, respiratory distress syndrome, and aging diseases (Ruffmann and Wendel, 1991). Given its role in mitigating tissue damage and its diverse therapeutic potential, we have been committed to exploring the effects and molecular mechanisms of hepatic protection and cerebral protection of GSH. Our previous data suggested that GSH could not only alleviate hepatotoxicity caused by doxorubicin, but also exert therapeutic effects on ischemic stroke by increasing intracerebral dopamine levels (Shen et al., 2019; Chen et al., 2020). Hepatic encephalopathy is a severe complication of acute and chronic liver diseases, accompanied by extensive neuropsychiatric abnormalities, including a series of defects in psychomotor, motor, cognitive, emotional and behavioral functions. Despite decades of research, the mechanisms underlying AHE remain poorly understood, and its clinical treatment regime is limited to liver transplantation. Due to the limitation of time constraints and donor availability, the mortality rate of AHE is up to 80% (Fu et al., 2019). Since GSH has both hepatic and cerebral protective effects, we speculate that GSH might be used to treat AHE. In this study, we aimed to systematically investigate the feasibility of GSH as a treatment for AHE through pharmacokinetic, pharmacodynamic, and mechanism studies.

Our findings demonstrated that exogenous GSH significantly improved behavioral scores and reduced hepatic damage in AHE rats with TAA-induced AHE, as evidenced by improvements in behavioral scores and reductions in markers of hepatic injury and inflammation. Additionally, we observed alterations in amino acid metabolism, particularly an imbalance in plasma amino acids, which are known to contribute to the pathogenesis of AHE (Holecek, 2015; Gluud et al., 2017). According to previous studies, in patients with hepatic encephalopathy, AAAs increased due to the reduction of liver capacity, and BCAAs reduced due to the activation of glutamine synthesis in muscle (Görg et al., 2015; Holecek, 2015). Notably, GSH administration helped maintain the balance of BCAAs and AAAs, suggesting its hepatoprotective effects.

Furthermore, we investigated the cerebral protective effect of exogenous GSH on AHE rats. Our data indicated that exogenous GSH significantly regulated inflammatory factors and reduced neuronal apoptosis, particularly in cortical regions. Recently, glutamine was considered as an intracerebral toxic substance that could lead to brain edema, which was directly involved in the pathogenesis of AHE (Lemberg and Fernández, 2009). Herein, we found that oral GSH also significantly attenuated the accumulation of glutamine by up-regulating the expression of GS enzymes that catalyze ammonia production of glutamine. Additionally, the liver and brain are the main organs responsible for taurine synthesis, which was implicated in K^+^ and Ca^2+^ homeostasis in the brain. In both acute and chronic liver failure, the concentrations of intracerebral taurine would decrease (Butterworth, 1996). Our results demonstrated that oral GSH administration significantly restored intracerebral taurine levels. Thus, exogenous GSH could exert significant cerebral protective effects on AHE rats.

Generally, extracellular GSH could be hydrolyzed to cysteine-glutathione (CYS-GLY) and glutamate (GLU) by the intestinal γ-glutamyl transferase, and the CYS-GLY could be further cleaved to generate CYS and GLY (Chen et al., 2020). Interestingly, most hydrolysates of GSH are related to various pathological and physiological processes (Luo et al., 2021). To reveal the pharmacological substances that GSH exerts therapeutic effects on AHE, the exposure of GSH-derived ingredients (GSH, CYS, GLU, GLY, and, CYS-GLY) in the target tissues was determined using LC-MS/MS and LC-Q-TOF/MS systems. Pharmacokinetic studies revealed that GSH administration increased GSH and CYS levels in target tissues, particularly in the cortex, highlighting its specific effects on brain tissues. Furthermore, we found that oral GSH could elevate the GSH levels in liver and intestines (colon and ileum), whereas intravenous administration cannot. The potential reasons might lie in low dose and high short half-life.

Oxidative stress is a common phenomenon in the body, often associated with various diseases (Simicic et al., 2022). In the case of AHE, increased oxidative stress is considered a key factor contributing to its pathogenesis. Elevated levels of blood and brain ammonium are also implicated in AHE, leading to inflammation, neurotoxicity, and oxidative stress (Görg et al., 2013; 2015). Despite its significance, the precise relationship between oxidative stress regulation and pathophysiology/treatment of AHE remains incompletely understood. Therefore, we attempted to reveal the molecular mechanism of GHS treatment for AHE by focusing on its regulation of oxidative stress. Our results suggested that AHE modeling led to a noticeable enhancement of ROS, and GSH administration effectively restored ROS levels to normal, indicating its ability to mitigate oxidative stress in AHE. The balance between GSH and its oxidized form GSSG is crucial in protecting cells from oxidative damage (Cao et al., 2013). Our data indicated that GSH administration significantly reduced the GSSG/GSH ratio in AHE rats,. Moreover, MDA, a commonly used biomarker of oxidative stress resulting from lipid peroxidation of polyunsaturated fatty acids (Khoubnasabjafari et al., 2015), was found to be elevated in AHE modeling but significantly reversed by GSH administration. Additionally, GSH administration effectively reversed the downregulation of SOD, an important antioxidant enzyme, in the cortex of AHE rats, further supporting its antioxidative properties. NADPH oxidases (NOXs), multi-subunit membrane-bound enzymes, could catalyze the reduction of oxygen into superoxide by using NADPH as an electron donor and oxygen as an electron acceptor (Zeynab et al., 2014). Among 7 NOX isoforms (NOX1, NOX2, NOX3, NOX4, NOX5, dual oxidase 1, and dual oxidase 2), NOX4 is a major isoform and plays a significant role in generating ROS in astrocytes (Serrander et al., 2007). Our results revealed that the AHE modeling significantly upregulated the expression of NOX4 in the rat cortex, while GSH administration reversed the upregulation of NOX4 expression, indicating its ability to suppress ROS production through NOX4 downregulation. Furthermore, NF-κB is a key regulator of inflammatory response and oxidative stress, often triggered by TNF-α (Aoki et al., 2021). Our results showed that GSH administration reversed the upregulation of NF-κB P65 expression in AHE rats, suggesting its role in mitigating inflammatory response and oxidative stress via NF-κB inhibition. In summary, our findings demonstrate that GSH administration significantly alleviates oxidative stress levels induced by AHE modeling by downregulating NOX4 and NF-κB P65, highlighting its potential as a therapeutic intervention for AHE.

Microglia, the primary immune cells usually remaining static in the CNS, play a pivotal role in responding to various stimuli and maintaining CNS homeostasis (Butturini et al., 2019). However, overactivation of microglia can lead to the release of proinflammatory cytokines and exacerbate neuronal damage, contributing to the pathogenesis of various neurodegenerative diseases (Zhang et al., 2019; Huang et al., 2022). Herein, we found that AHE modeling induced microglia activation, which was significantly inhibited by GSH administration. Generally, microglia activation is mainly categorized into two phenotypes M1 and M2. The M1 activation state leads to the release of pro-inflammatory cytokines, and the genes related to M1 activated microglia mainly include iNOS, TNF-α, and IL-1β (He et al., 2021). Our findings revealed that AHE modeling significantly upregulated the expression of these M1 microglia-related genes, indicating neuroinflammation. However, GSH administration effectively reduced the expression of iNOS, TNF-α, and IL-1β, and variation rate of iNOS was the highest. iNOS, a key enzyme in the production of nitric oxide (NO), plays a crucial role in neuroinflammation and oxidative stress. Inhibition of iNOS with L-NAME enhanced cell viability in NH4Cl-simulated U251 cells, further implicating its involvement in AHE pathogenesis. Consistently, we found that GSH administration reduced the abnormal elevation of NO induced by AHE modeling in the cortex. Furthermore, ER stress can induce apoptosis or inflammation through one or more of the three pathways, mainly including PERK-ATF4, IRE1-XBP1s or ATF6 (Soni et al., 2023). Given the association between iNOS and ER stress-induced apoptosis, we measured ER stress-related genes (ATF4, IRE1α, and ATF6) to further verify the crucial role of iNOS in AHE. Our results revealed that GSH administration could exert a therapeutic role in AHE by regulating ER stress through iNOS/ATF4/Ddit3 pathway, suggesting its therapeutic potential in mitigating ER stress-induced apoptosis and inflammation in AHE.

## 5 Conclusion

In this study, we systematically explored the feasibility of GSH in treating AHE through pharmacokinetic, pharmacodynamic, and mechanistic studies. We found that both oral and intravenous GSH significantly reduced TAA-induced blood ammonia accumulation and brain damage, with only oral GSH effectively reducing liver injury. Oral GSH also regulated amino acid homeostasis, decreased intracerebral glutamine and GS accumulation, and increased taurine exposure. Pharmacokinetic analysis revealed reduced GSH and CYS levels in the liver and brain during AHE modeling, which were restored by both oral and intravenous GSH, while only oral GSH restored intrahepatic GSH and CYS levels, potentially explaining its efficacy in liver injury inhibition. Additionally, AHE modeling and oral GSH notably affected GSH and CYS levels in the cortical region, consistent with the prominent anti-inflammatory effect of GSH in the cortex. Furthermore, oral GSH downregulated NOX4/NF-κB P65 expression, inhibited microglia activation and M1 microglia-related gene expression, particularly iNOS, and regulated ER stress via the iNOS/ATF4/Ddit3 pathway, demonstrating its therapeutic mechanism in AHE.

## Figures and Tables

**Table:** 

**AAA**	aromatic amino acid
**AHE**	acute hepatic encephalopathy
**ALF**	acute liver injury
**BCAA**	branched-chain amino acid
**CAP**	captopril
**CNS**	central nervous system
**CYS**	cysteine
**GADPH**	glyceraldehyde 3-phosphate dehydrogenase
**ER**	endoplasmic reticulum
**Glu**	glutamate
**GLY**	glycine
**GS**	glutamine synthetase
**GSH**	Glutathione
**GSSG**	L-Glutathione Oxidized
**H&E**	hematoxylin-eosin
**IL-1β**	interleukin-1β
**IL-6**	interleukin-6
**IL-10**	interleukin-10
**iNOS**	inducible nitric-oxide synthase
**L-NAME**	N(ω)-nitro-L-arginine methyl ester
**MDA**	malonic dialdehyde
**MRM**	multiple reaction monitoring
**MSI**	Mass Spectrometry Imaging
**NADPH**	nicotinamide adenine dinucleotide phosphate
**NEM**	N-ethylmaleimide
**NMDA**	N-methyl-D-aspartic acid
**ROS**	reactive oxygen species
**SOD**	superoxide dismutase
**TAA**	thioacetamide
**TNF-α**	tumor necrosis factor-alpha

## Data Availability

The raw data supporting the conclusion of this article will be made available by the authors, without undue reservation.
