# Erratum: Feasibility exploration of GSH in the treatment of acute hepatic encephalopathy from the aspects of pharmacokinetics, pharmacodynamics, and mechanism

**DOI:** 10.3389/fphar.2024.1534991

**Published:** 2024-12-09

**Authors:** Frontiers Production Office

**Affiliations:** Frontiers Media SA, Lausanne, Switzerland

**Keywords:** glutathione, acute hepatic encephalopathy, oxidative stress, endoplasmic reticulum stress, iNOS/ATF4/Ddit3

Due to a production error, an incorrect number was provided for “the National Natural Science Foundation of China”. The correct number is 82274194.

The publisher apologizes for this mistake. The original version of this article has been updated.

