# Supplementary material for: Feasibility exploration of GSH in the treatment of acute hepatic encephalopathy from the aspects of pharmacokinetics, pharmacodynamics, and mechanism
Source: Front Pharmacol. 2024 Jun 3;15:1387409. doi: 10.3389/fphar.2024.1387409 (PMC11181355; doi:10.3389/fphar.2024.1387409)
Supplement: Supplementary file 1 [file DataSheet1.DOCX]

**Captions of Supplemental Data**

**Fig. S1.** Influence of oral administration of GSH on the distribution of GLU, GLY, and CYS-GLY in the brain and liver of AHE rats. Intrahepatic levels of (A) CYS-GLY, (B) GLY, (C) GLU; Intracerebral levels of (D) CYS-GLY, (E) GLY, (F) GLU.

**Fig. S2.** Influence of intravenous administration of GSH on the distribution of GSH and CYS in the brain and liver of AHE rats. (A) Intrahepatic GSH, (B) Intrahepatic CYS, (C) GSH in cortex, (D) CYS in cortex, (E) GSH in hippocampus, (F) CYS in hippocampus, (G) GSH in striatum, (H) CYS in striatum.

**Fig. S3.** Influence of intravenous administration of GSH on the distribution of GSH in the tissues of AHE rats. Exposure of GSH in (A) heart, (B) kidney, (C) duodenum, (D) jejunum, (E) ileum, (F) colon**.**

**Table S1** MRM monitoring parameters of GSH-derived components and internal standard for LC-MS/MS determination.

**Table S2** MRM monitoring parameters DESI-MSI analysis.

**Table S3** MRM monitoring parameters of AAs and internal standard for LC-MS/MS determination.

**Table S4** Q-PCR primer sequences used in this study.


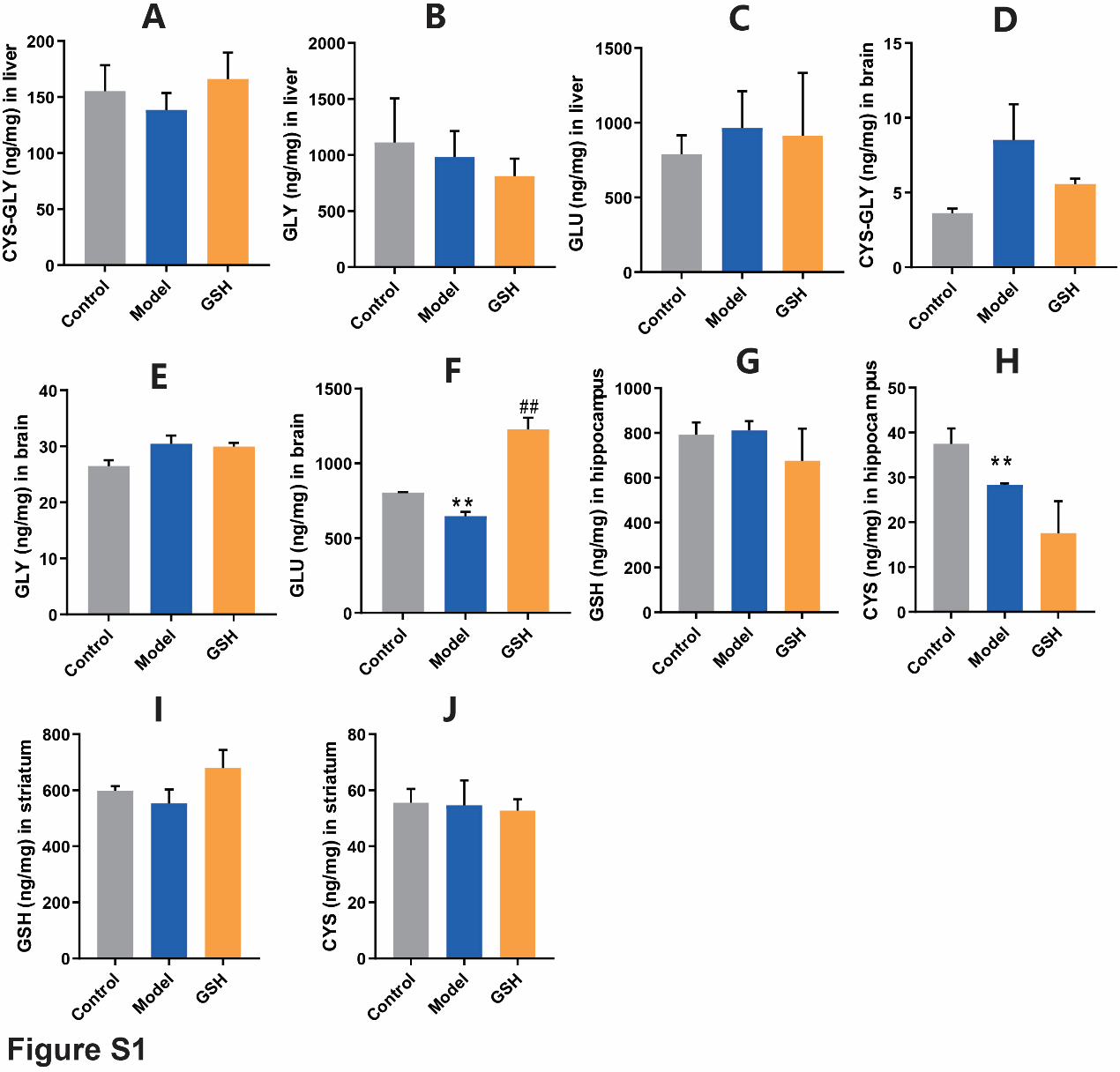


**Fig. S1.** Influence of oral administration of GSH on the distribution of GSH, CYS, GLU, GLY, and CYS-GLY in the brain and liver of AHE rats. Intrahepatic levels of (A) CYS-GLY, (B) GLY, (C) GLU; Intracerebral levels of (D) CYS-GLY, (E) GLY, (F) GLU; levels of (G) GSH and (H) CYS in striatum; levels of (I) GSH and (J) CYS in hippocampus.

**
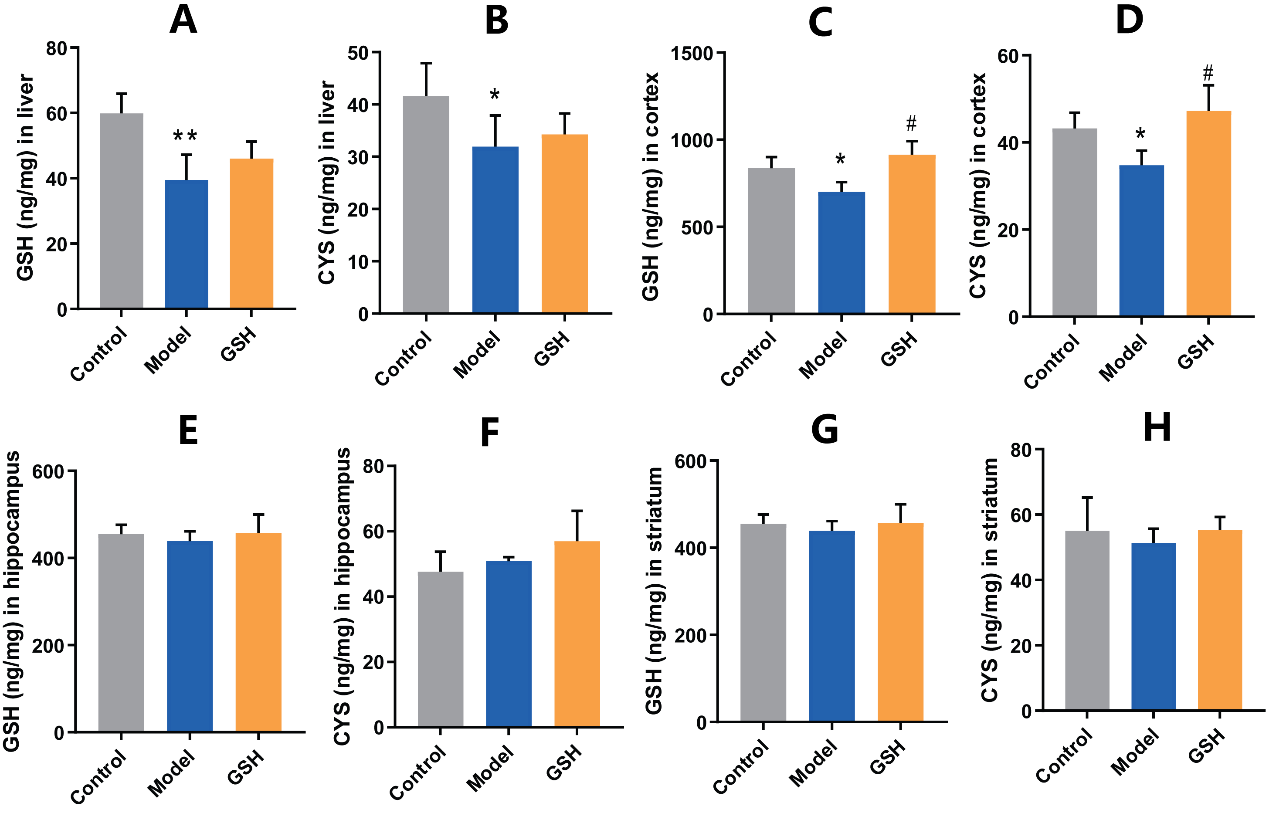
**

**Fig. S2.** Influence of intravenous administration of GSH on the distribution of GSH and CYS in the brain and liver of AHE rats. (A) Intrahepatic GSH, (B) Intrahepatic CYS, (C) GSH in cortex, (D) CYS in cortex, (E) GSH in hippocampus, (F) CYS in hippocampus, (G) GSH in striatum, (H) CYS in striatum.

**
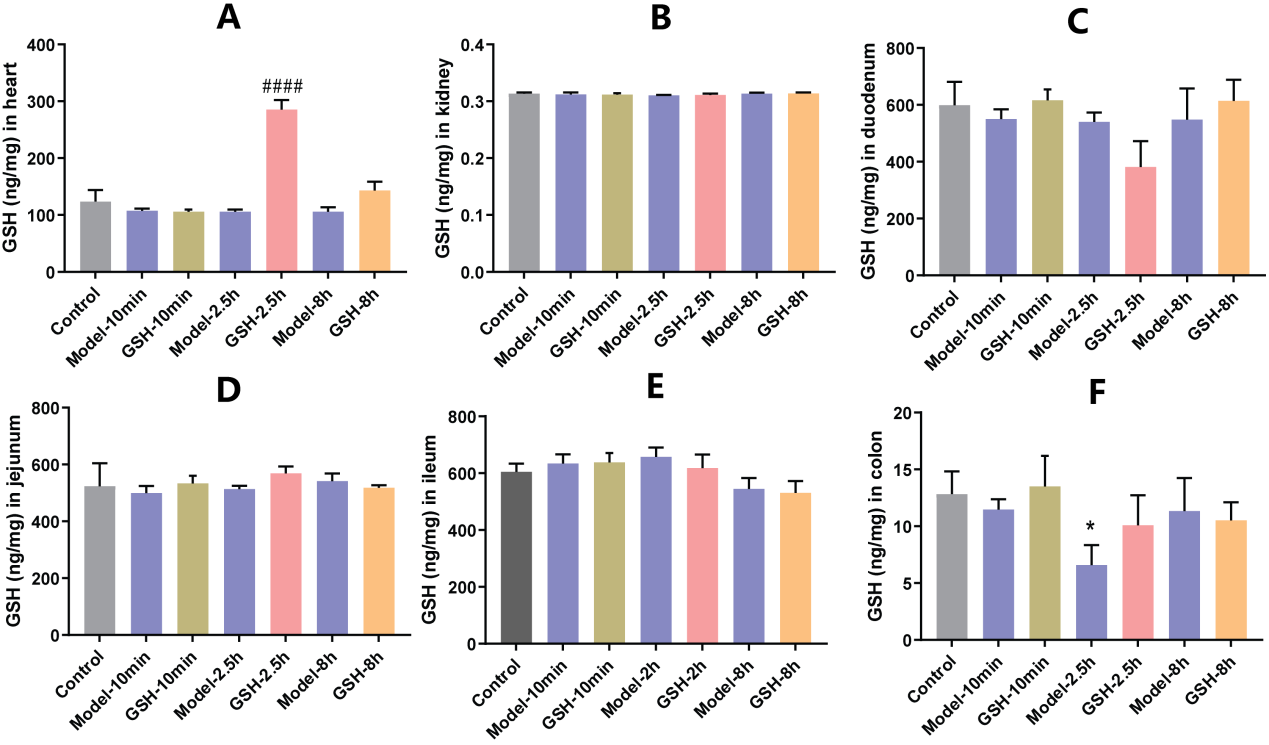
**

**Fig. S3.** Influence of intravenous administration of GSH on the distribution of GSH in the tissues of AHE rats. Exposure of GSH in (A) heart, (B) kidney, (C) duodenum, (D) jejunum, (E) ileum, (F) colon**.**

**Table S1** MRM monitoring parameters of GSH-derived components and internal standard for LC-MS/MS determination.

| **Analyte** | **Precursor**  **（*m/z*）** | **Product**  **（*m/z*）** | **DP**  **(V)** | **CE**  **(eV)** |
| --- | --- | --- | --- | --- |
| GSH-NEM | 433.1 | 304.1 | 100 | 20 |
| CAP-NEM | 343.1 | 228.1 | 100 | 30 |
| CYS-NEM | 246.9 | 158.2 | 100 | 30 |
| CYS-GLY-NEM | 304 | 287.1 | 100 | 30 |

**Table S2** MRM monitoring parameters DESI-MSI analysis.

| **Analyte** | **Parent**  **（*m/z*）** | **Daughter**  **（*m/z*）** | **Cone**  **(V)** | **Collision**  **(eV)** | **Polarity** |
| --- | --- | --- | --- | --- | --- |
| GSH | 306 | 143 | 20 | 25 | Negative |
| CYS | 122 | 77 | 20 | 20 | Positive |
| Gln | 145 | 74 | 20 | 20 | Negative |
| Tau | 124 | 80 | 20 | 20 | Negative |

**Table S3** MRM monitoring parameters of AAs and internal standard for LC-MS/MS determination.

| **Analyte** | **Precursor**  **（*m/z*）** | **Product**  **（*m/z*）** | **DP**  **(V)** | **CE**  **(eV)** |
| --- | --- | --- | --- | --- |
| Ala | 194 | 105 | 30 | 10 |
| Arg | 279 | 105 | 60 | 15 |
| Leu | 236 | 105 | 55 | 15 |
| Lys | 355 | 188 | 60 | 5 |
| Met | 254 | 105 | 70 | 10 |
| Phe | 270 | 120 | 45 | 10 |
| Pro | 220 | 105 | 90 | 10 |
| Tyr | 390 | 105 | 45 | 10 |
| Val | 222 | 105 | 70 | 10 |
| Ser | 210 | 105 | 60 | 10 |
| Thr | 224 | 105 | 55 | 10 |
| Glu | 252 | 105 | 60 | 15 |
| Asn | 237 | 105 | 60 | 15 |
| Asp | 238 | 105 | 60 | 15 |
| Gln | 251 | 105 | 65 | 10 |
| Trp | 309 | 263 | 75 | 10 |
| Gly | 180 | 105 | 80 | 10 |
| DHB | 380 | 105 | 45 | 20 |

**Table S4** Q-PCR primer sequences used in this study.

| **Primer** | **Forward Primer Sequence** | **Reverse Primer Sequence** |
| --- | --- | --- |
| IL-6 | GGCAGGTCTACTTTGGAGTCATTGC | ACATTCGAGGCTCCAGTGAATTCGG |
| IL-1β | CAAAGCCAGAGTCCTTCAGAG | GTCCTTAGCCACTCCTTCTG |
| TNF-α | GTCCTTAGCCACTCCTTCTG | CAGGACAGGTATAGATCTTTCCTTT |
| GS | TCCAGATAGGACCCTGCGAA | GCTAAAGTTGGTGTGGCAGC |
| β-actin | TCAGGTCATCACTATCGGCAAT | AAAGAAAGGGTGTAAAACGCA |
| iNOS | TGGGTCTTGTTAGCCTAGT | TCACCTTGGTAGGATTTGAC |
| Arg-1 | CCGCAGCATTAAGGAAAGC | CCCGTGGTCTCTCACATTG |
| YM-1 | AGTCATCAAATTCCTTCGCCAGTAT | GCCTTGGGATTTCTTGCTCAGT |
| CD206 | CTCTAAGCGCCATCTCCGTT | ATGATCTGCGACTCCGACAC |
| ATF-4 | ACCAGTCGGGTTTGGGGGCT | TTCCGAGGAGCCCGCCTTGT |
| ATF-6 | GATTTGATGCCTTGGGAGTC | GGACCGAGGAGAAGAGACAG |
| Ddit3 | ACGGAAACAGAGTGGTCAGT | AGACAGACAGGAGGTGATGC |
| IRE1α | TGGACGGACAGAATACACCA | TGGACACAAAGTGGGACATC |
| Xbp1 | GATGAATGCCCTGGTTACTG | AGATGTTCTGGGGAGGTGAC |
| GAPDH | AGGATACTGAGAGCAAGAGA | TTGATGGTATTCGAGAGAAGG |
